# American Cranberry (*Oxycoccus macrocarpus* (Ait.) Pursh) Leaves Extract and Its Amino-Acids Preparation: The Phytochemical and Pharmacological Study

**DOI:** 10.3390/plants12102010

**Published:** 2023-05-17

**Authors:** Oleh Koshovyi, Inna Vlasova, Valdas Jakštas, Gabrielė Vilkickytė, Vaidotas Žvikas, Roman Hrytsyk, Lyubov Grytsyk, Ain Raal

**Affiliations:** 1 Institute of Pharmacy, Faculty of Medicine, University of Tartu, Nooruse 1, 50411 Tartu, Estonia; 2 Department of Pharmacognosy, The National University of Pharmacy, 53 Pushkinska St, 61002 Kharkiv, Ukraine; innavlasova.ukraine@gmail.com; 3 Institute of Pharmaceutical Technologies, Lithuanian University of Health Sciences, 44307 Kaunas, Lithuania; valdas.jakstas@lsmu.lt (V.J.); gabriele.vilkickyte@lsmu.lt (G.V.); vaidotas.zvikas@lsmu.lt (V.Ž.); 4 Department of Pharmaceutical Management, Drug Technology and Pharmacognosy, Ivano-Frankivsk National Medical University, 2 Halytska Str., 76018 Ivano-Frankivsk, Ukraine; grytsyk95@gmail.com (R.H.); grycyk_l@ukr.net (L.G.)

**Keywords:** American cranberry (*Oxycoccus macrocarpus*), leaves, extract, arginine, valine, hepatoprotective activity

## Abstract

The liver is an organ with several important biofunctions, for which there are very few effective and safe preparations that promote the functioning, protection, and regeneration of cells. Sufficiently safe preparations with hepatoprotective activity can be found in plants. The aim of our study was to investigate the chemical composition of an extract made from American cranberry (*Oxycoccus macrocarpus* (Ait.) Pursh) leaves and its amino-acids preparations as well as their possible hepatoprotective activity. Using the UPLC-MS/MS method, we identified 19 phenolic compounds (8 flavonoids (flavones and flavonols), 4 anthocyanins, 3 hydroxycinnamic acids, and 2 catechins). The prophylactic and therapeutic administration of the American cranberry-leaves extracts led to a decrease in the lipid-peroxidation process during a study of tetrachloromethane acute toxic damage in the liver of rats. As a result of animal studies, the most effective hepatoprotective activity was found in the extract preparations with valine and arginine.

## 1. Introduction

The liver performs a fundamental role in the regulation of diverse physiological processes and biofunctions. [1,2,3]. Amongst the gastrointestinal manifestations experienced by COVID-19 patients, those commonly noted were diarrhea, anorexia, nausea, vomiting, and abdominal pain. Hepatic injury was evident in some patients, the degree of which at times can mirror the severity of the disease; pancreatic injury had been noted as well [4,5].

Although the liver has a high regenerative capacity, endogenous and exogenous stimuli can still result in permanent tissue damage and impairment of liver function [6,7]. Hepatic diseases can damage the cells, tissues, structure, or liver function [8,9]. There do not exist effective drugs to stimulate hepatic functioning [10]. Thus, it is important to find alternative preparations from plants for the treatment of hepatic diseases, with the aim of these agents being more effective and less toxic.

The consumption of berry extracts has increased in dietary supplements and functional foods. The most commonly used berries are black raspberries (*Rubus occidentalis*), blackberries (*Rubus* spp.), strawberries (*Fragaria X ananassa*), red raspberries (*Rubus idaeus*), cranberries (*Vaccinium macrocarpon*), and blueberries (*Vaccinium corymbosum*) [11]. These fruits have significant antioxidative activity. Dysfunction of cellular immunity and oxidative stress are specific indicators of hepatic disease pathogenesis [12]. It was noticed that blueberries affect liver protection and cellular immune function [13].

According to the data [1], different berries are consumed frequently, and among these, is the cranberry. This experiment evaluated its hepatoprotective and antioxidant potential against liver mitochondrial damage induced by CCl4 intoxication in rats. The administration of cranberries was effective in diminishing the CCl4 toxic effects, normalizing alanine aminotransferase (AlAt), aspartate aminotransferase (AsAt) activity, and bilirubin concentrations. Moreover, it prevented the accumulation of membrane lipid peroxidation products in the liver, which resulted in the preservation of the mitochondrial ultrastructure [14].

The fruits and compounds mentioned [1] could give novel perspectives to the limited therapeutic effects for the treatment of liver diseases. In general, biologically active substances and extracts from cranberries provided hepatoprotective effects, which mechanisms are related to antioxidant activity [15].

Being cultivated, cranberry bushes are annually pruned, so tons of leaves are wasted. These leaves contain biologically active substances and could be further used as a source of plant-origin products. From this point of view, *Oxycoccus macrocarpus* (Ait.) leaves (Pursh, *Ericaceae*) are interesting for the creation of health products with hepatoprotective activity.

Previous studies have shown that plants in the *Vaccinium* genus are promising sources for the creation of hypoglycemic and hypolipidemic agents because of their significant antioxidant activity, namely extracts from bilberry leaves [16], blueberry leaves [17] and bearberry leaves [18,19]. Studies have shown that extracts from the leaves of cranberries are also promising when used to correct insulin-resistant conditions [20,21].

The modification of molecules with amino acids is a well-known strategy for modeling their biological activity. Thus, the antiviral medicine Valtrex was created by synthesizing acyclovir and valine [22], and L-lysine escinate—the angioprotective drug—was developed by combining chestnut triterpene saponins complex (β-escin) with L-lysine [22,23]. Motherwort tincture was modified with amino acids and resulted in the development of a new active anxiolytic medicine [24]. The modification of a blueberry-leaves extract with arginine led to the creation of a new remedy with pronounced hypolipidemic and hypoglycemic effects [16]. The combination of phenylalanine with a bearberry-leaves extract allowed the researchers to obtain a product with anti-inflammatory and pronounced diuretic activity [18,19] and with cysteine—a new one for insulin resistance management [25].

Thus, the aim of our investigation was to study the qualitative and quantitative content of phenolics in American cranberry-leaves extracts and their hepatoprotective activity in combination with amino acids. We are the first to combine the active ingredients of cranberry leaves with amino acids and to demonstrate their hepatoprotective effects.

## 2. Results

### 2.1. HPLC Analysis and Quantification of Major Compounds

**Figure 1:**
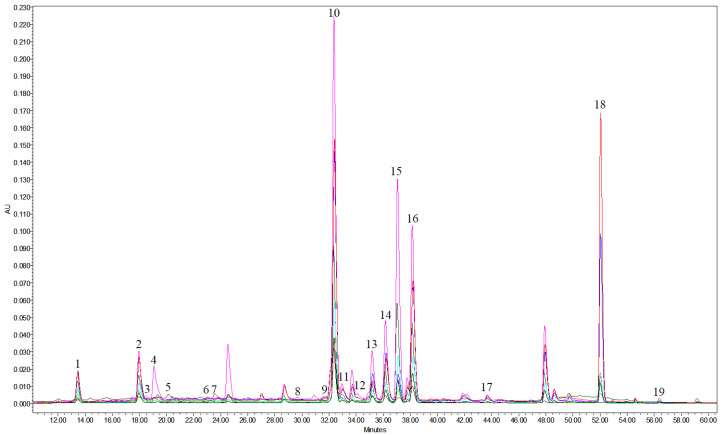
HPLC-PDA profiles of phenolics in cranberry-leaves extracts at 325 nm. Peak assignments: 1—neochlorogenic acid, 2—chlorogenic acid, 3—(+)-catechin, 4—4-*O*-caffeoylquinic acid, 5—cyanidin-3-*O*-galactoside, 6—(–)-epicatechin, 7—cyanidin-3-*O*-arabinoside, 8—procyanidin A1, 9—*p*-coumaric acid, 10—hyperoside, 11—isoquercitrin, 12—procyanidin A2, 13—reynoutrin, 14—quercetrin-3-*O*-arabinopyranoside, 15—avicularin, 16—quercitrin, 17—kaempferol-3-O-rhamnoside, 18—quercetin, 19—kaempferol.

The major phenolic compounds of the cranberry-leaves extracts were identified by UPLC-MS/MS and HPLC-PDA methods. Characteristic phenolic compounds (cyanidin-3-*O*-galactoside, 3-*O*-arabinoside, hyperoside, isoquercitrin, reynoutrin, quercetin-3-O-arabinopyranoside, avicularin, quercitrin, quercetin, kaempferol-3-*O*-rhamnoside, kaempferol, chlorogenic, neochlorogenic, 4-*O*-caffeoylquinic acid, *p*-coumaric acid, (+)-catechin and (–)-epicatechin, procyanidin A1, A2, B1, and B2) were found. Typical MRM chromatograms are presented in Figure 1. Chromatograms and qualitative results of the HPLC-PDA method are presented in Table 1 and in Figure 1 and Figure S2, while typical UPLC-MS/MS chromatograms of phenolic components in the samples are given in Figure S1.

### 2.2. Acute Toxicity

In the control pathology group, 2 animals died (1 animal on the 1st day and 1 animal on the 2nd day of the experiment). Thus, mortality in the group of control pathology was 33%. In the other groups, all animals remained alive until the end of the experiment, which indicates the prospects for research into the hepatoprotective activity of the studied extracts.

### 2.3. Hepatoprotective Activity

The cranberry-leaves extracts show hepatoprotective activity in the acute-tetrachloromethane-hepatitis model. The fact was confirmed by morphological and biochemical indicators. The results of measuring liver weight coefficients (LWC) for the experimental animals are given in Table 2. In the experiment, it was noticed that in the animals in the control pathology group, the increase in liver weight was 45.17% compared to the intact animals, which indicates liver damage and inflammation. After taking the American cranberry-leaves extracts, according to LWC, animals in the groups that received the combined extracts E2 and E3 at a dose of 25 mg/kg (bw) were similar to the intact animals, while the decreases in liver weight were 27.2% and 25.64%, respectively, compared to the control pathology group. The animals treated with the referent drug “Silibor” had a decrease of 22.5% in liver weight compared to the control pathology group. The consumption of the other studied extracts (E6, E8, E7, E5, E4, E1) caused decreases of 21.92%, 20.74%, 20.16%, 19.37%, 18.40%, 17.80%, respectively, in liver weight, which is comparative with the values in the animals taking the referent drug “Silibor”.

The results of measuring biochemical indicators in blood serum and liver homogenate, such as alanine aminotransferase, aspartate aminotransferase, and 2-thiobarbituric acid reactants are presented in Table 3. The results of biochemical indicators studies show that a single consumption of tetrachloromethane led to the development of acute toxic liver damage. In the control pathology group, significant intensification of lipid-peroxidation processes and the exhaustion of the antioxidant protection system were observed, as a result of which, the structural and functional integrity of the membranes was violated. The destruction of cell membrane components led to the development of a pronounced cytolytic syndrome, as evidenced by a 3.37-times increase in AlAt activity in blood serum and by a 2.42-times increase in AsAt activity, compared to the parameters of the intact group. The development of acute toxic hepatitis was characterized by an increase in peroxide catabolic transformations, as evidenced by an increase in the content of TBA-reactants in blood serum and liver homogenates of untreated animals by 1.85 times and 2.51 times, respectively, compared to the indicators of the intact animals.

## 3. Discussion

### 3.1. Phytochemical Research

The researchers identified 19 phenolic substances in the American cranberry-leaves extracts, and their contents were determined by the HPLC method. There were eight flavones and flavonols, four anthocyanins, three hydroxycinnamic acids, and two catechins. Quercetin glycosides predominate in the extracts. Hyperoside, avicularin, and quercitrin were dominant among the individual substances (Table 1).

The phenolic profile of cranberry leaves and fruits is rather different. Cranberries contain three classes of flavonoids, such as anthocyanins, flavonols, and proanthocyanidins [26]. The predominate flavonoids in cranberries are the glycosides of quercetin and myricetin: quercetin-3-glucoside, quercetin-3-galactoside, quercetin-3-rhamnospyranoside, quercetin-3-O-(6′′-p-benzoyl)-galactoside, myricetin-3-galactoside, and myricetin-3-arabinofuranoside [27,28].

Both American cranberries and European cranberries accumulate a high level of valuable phenolics. European cranberries contain higher amounts of caffeic acid, quercetin, and flavonols than American cranberries [28,29,30]. The differences in the composition of polyphenolics in cranberries may be affected by various conditions such as weather, maturity stage, cultivation, harvesting, region, etc. [31].

It was noticed that the content of aglycones, such as quercetin and kaempferol, increases after the extract E1 modification with amino acids. It indicated that the hydrolysis of glycosides was taking place. These could be detected in the extracts E6 and E8 for kaempferol and in the extracts E2, E5, E6, E7, and E8 for quercetin.

The content of glycosides decreased in the extract E1 preparations with amino acids almost all of the time. This occurred due to the addition of amino acids and the possible formation of their conjugates with these substances.

The content of hydroxycinnamic acids in the modified extracts (E2-E8) was significantly lower. This can be explained by creating conjugates with amino acids, which confirms previous research on highbush blueberry extracts [17].

### 3.2. Hepatoprotective Activity

The results of the research (Table 2) show that the consumption of hepatotropic poison CCl4 led to a significant increase in LWC, which indicates liver damage. In the experimental groups that took the American cranberry-leaves extracts and the referent drug “Silibor”, the changes were insignificant compared to the intact animals. The best corrective effect was shown by the combined extracts (E2 and E3) with arginine and valine, which were more effective than the referent drug “Silibor”. The other studied extracts show activity at the level of the referent drug “Silibor” (E6, E7, and E8) or slightly lower (E1, E2, E5).

Thus, the LWC values indicate a positive effect of the American cranberry-leaves extracts and the referent drug “Silibor” and a decrease in swelling and normalization of blood circulation in the organ and, therefore, a decrease in the intensity of the inflammatory process.

The ingestion of the studied American cranberry-leaves extracts and the referent drug “Silibor” in experimental hepatitis studies under the conditions of a therapeutic and preventive regimen was accompanied by a noticeable decrease in pathological manifestations and led to a significant decrease in the studied indicators relative to the values in the control pathology group. The referent drug “Silibor” led to a decrease in the activity of the studied enzymes in the blood serum of experimental animals relative to the values in the control pathology group, namely AlAt by 2.02 times and AsAt by 1.87 times.

When the American cranberry-leaves extract (E1) was given to the animals at a dose of 25 mg/kg (bw), the activity of biochemical indicators in blood serum decreased relative to the values in the control pathology group: AlAt by 2.0 times and AsAt by 1.71 times, which was at the level of the indicators in the group taking the reference drug “Silibor”.

Adding amino acids to the extract E1 led to an increase in their hepatoprotective activity. The best effect was shown by the extracts with amino acids valine (E2) and arginine (E3), which had a high impact on the development of cytolysis syndrome, reducing the activity of AlAt by 3 times and 2.67 times, and AsAt by 2.23 times and 2.13 times, respectively, relative to the values in the control pathology group. These extracts were more active than the referent drug “Silibor” and brought the biochemical indicators of blood serum to the level of the intact animals.

The extracts with amino acids histidine (E6) and taurine (E8) showed slightly better activity compared to the group of animals treated with the referent drug “Silibor”. At the same time, the activity of AlAt was 2.42 times and 2.33 times lower, and AsAt was 2.0 times and 2.07 times lower in comparison with the control pathology group.

The studied extracts with aspartic acid (E7) and glycine (E5) had activity at the level of the referent drug “Silibor”, and the extract with alanine (E4) did not lead to an improvement in the indicators of the antioxidant system in comparison with the group of animals treated with the referent drug “Silibor”.

Simultaneous administration of hepatotropic poison and the American cranberry-leaves extracts (E1, E3, E2, E6, E8, E7, E5, and E4) at a dose of 25 mg/kg (bw) resulted in a decrease in the level of TBA-reactants in blood serum by 1.42, 1.76, 1.69, 1.61, 1.57, 1.50, 1.48, and 1.43 times, respectively, and in the liver homogenate by1.69, 2.23, 2.12, 1.90, 1.98, 1.68, 1.62, and 1.63 times, respectively. The use of the referent drug “Silibor” led to a decrease in the level of TBA-reactants in blood serum and liver homogenate by 1.53 and 1.72 times, respectively. A significant decrease in the activity of all studied enzymes in blood serum and liver homogenate indicates a positive effect against hepatocytes cytolysis.

The most significant hepatoprotective effect has been detected for the extracts prepared with valine and arginine, which with a dose of 25 mg/kg (bw) authentically exceeded in activity the referent drug “Silibor” on such indicators as LWC, the activity of AlAt and AsAt enzymes, and the level of lipid peroxidation of TBA-reactants and brought their blood and liver homogenate to the level of the intact animals. The use of American cranberry-leaves extract and its preparations with histidine, taurine, aspartic acid, glycine, and alanine did not significantly lead to an increase in hepatoprotective activity. Biochemical indicators of blood serum and liver homogenate were at the same level or slightly lower than the referent drug “Silibor”.

All studied extracts of the American cranberry leaves can be arranged in the following sequence according to the level of hepatoprotective activity: E3 (arginine) → E2 (valine) → E6 (histidine) → E 8 (taurine) → E7 (aspartic acid) → E5 (glycine) → E4 (alanine) → E1.

Thus, the obtained results indicate that in conditions of acute toxic hepatitis caused by tetrachloromethane, the American cranberry-leaves extracts have hepatoprotective activity, inhibiting peroxide destructive processes and reducing the development of cytolysis syndrome. The American cranberry-leaves extracts prepared with arginine and valine have a more pronounced hepatoprotective effect compared to the referent drug “Silibor”.

## 4. Materials and Methods

### 4.1. Plant Material

American cranberry leaves were harvested in August 2020 in the Kyiv region (Pereyaslav suburbs, 50.10314334026342, 31.46151900698126). The identity of the plants was established by Professor Tetiana Gontova, D.Sc. in Pharmacy [21,32]. Voucher specimens were deposited at the Pharmacognosy Department (National University of Pharmacy, Kharkiv, Ukraine, # 592–594). The raw material was dried at room temperature in a well-ventilated area for ten days and stored in paper bags. The raw material was standardized according to the proposed requirements [21,33,34].

### 4.2. Extracts Preparation

A total of 500.0 g of dried cranberry leaves [17,21,33] (size of particles 1–2 mm) were macerated with 3 L of 50 % aqueous ethanol solution in an extractor at room temperature overnight. The process was repeated three times (1.0 L of the same solvent). The extracts were combined, settled for two days, and filtered. The content of phenolic substances in the liquid cranberry extract in terms of gallic acid was 1.04%. Next, the obtained extract was divided into 8 parts. The first one was evaporated to dryness (E1).

To the other (500 mL each) amino acids: 10.5 g of valine (E2), 15.71 g of arginine (E3), 8.03 g of alanine (E4), 6.77 g of glycine (E5), 14.00 g of histidine (E6), 11.98 of aspartic acid (E7), and 11.41 g of taurine (E8) were added thrice at the equimolar quantity to phenolic compounds (according to established equivalents of gallic acid). More details have been described in an earlier study [35,36].

The extract E1 from the cranberry leaves is a brown loose powder with a specific smell. When it is modified with amino acids, its appearance changes: pink-red shades appear, which indicates the presence of anthocyanins in extract E1. The extracts E3, E4, E7, and E8 are viscous masses. The extracts E2, E5, and E6 remain powders.

### 4.3. Chemicals

The solvents used in the research were analytical grade acetonitrile, MS grade formic acid, and MS grade trifluoracetic acid from Merck (Darmstadt, Germany), acetonitrile from Sigma-Aldrich (Steinheim, Germany), and 96.0% ethanol from AB Vilniaus Degtine (Vilnius, Lithuania). The ultrapure water was purified by Milli–Q^®^ (Millipore, Bedford, MA, USA) water purification system.

Standard substances of neochlorogenic acid (5-*O*-caffeoylquinic acid), chlorogenic acid (3-*O*-caffeoylquinic acid), (+)-catechin, cryptochlorogenic acid (4-*O*-caffeoylquinic acid), (–)-epicatechin, procyanidins A1, A2, B1, and B2, p-coumaric acid, isoquercitrin (quercetin-3-*O*-glucoside), guaiaverin (quercetin-3-*O*-arabinopyranoside), avicularin (quercetin-3-*O*-arabinofuranoside), quercitrin (quercetin-3-*O*-rhamnoside), afzelin (kaempferol-3-*O*-rhamnoside), taurine, 17 amino acid mix solution, quercetin, and kaempferol were purchased from Sigma-Aldrich; hyperoside (quercetin-3-*O*-galactoside), cyanidin-3-*O*-galactoside, and cyanidin-3-O-arabinoside were obtained from Extrasynthese (Genay, France).

### 4.4. Analysis and Quantification of Major Compounds

#### 4.4.1. Phenolic Compounds Identification by UPLC-MS/MS

The analysis of phenolic compounds in selected samples was carried out with an Acquity H-class UPLC system (Waters, USA) equipped with a triple quadrupole tandem mass spectrometer (Xevo, Waters, USA). An electrospray ionization source (ESI) was used to obtain MS/MS data. YMC Triart C18 (100 × 2.0 mm 1,9 µm) column was used for the phenolic compounds separation. The temperature of the column was maintained at 40 °C. Gradient elution was performed using a mobile phase consisting of 0.1% formic acid aqueous solution (solvent A) and acetonitrile (solvent B) with a flow rate of 0.5 mL/min. A linear gradient profile was used with the following proportions of solvent A: 0 to 1 min—95%, 5 min.—70%, 7 min. 50%, 7.5 to 8 min. 0%, and 8.1 to 10 min. 95%. Negative electrospray ionization was applied for analysis with the settings: capillary voltage—2 kV, desolvation gas flow—700 l/h, cone gas flow—20 l/h, source temperature –150 °C, and desolvation temperature –400 °C. Identification and peak assignment of phenolic compounds in the cranberry extracts were based on a comparison of their retention times and MS/MS spectral data with those of standard compounds [37].

#### 4.4.2. Phenolic Compounds Analysis by HPLC-PDA

Qualitative and quantitative analysis of phenolic compounds was carried out on an HPLC-PDA (Waters e2695 Alliance system, Waters, Milford, MA, USA) system coupled with an ACE Super C18 (250 mm × 4.6 mm, 3 µm) reversed-phase column (ACT, Aberdeen, UK) with column temperature set at 35 °C according to the method reported earlier [37]. The mobile phase delivered at 0.5 mL/min consisted of 0.1% trifluoroacetic acid (eluent A) and acetonitrile (eluent B) with gradient elution: 0 min, 90% A; 0–40 min, 70% A; 40–60 min, 30% A; 60–64 min, 10% A; 64–70 min, 90% A. An injection volume of 10 µL was used. Identification and peak assignment of phenolics compounds in the cranberry extracts were based on a comparison of their retention times and absorption spectral data with commercial reference substances. The linear regression models were determined using the standard dilution method for quantification of compounds.

### 4.5. Acute Toxicity

Research in acute toxicity is mandatory in preclinical studies for new medicines. The method of determining the safety of a medicine used in studies [38,39,40] has been used to study the American cranberry leaves dry-extract acute toxicity.

The research was carried out on white outbred mice of both sexes at the vivarium of Ivano-Frankivsk National Medical University (IFNMU). They weighed 18–22 g and were on a regular diet. Groups of 6 animals were used in the experiment. They were given the cranberry-leaves extracts aqueous solution. Additionally, we used a control group. The solutions were consumed intragastrically in increasing doses with a metal probe.

The mice were observed for two weeks. The activity of the extracts was evaluated by integral indicators (body temperature, general and skin condition, changes in body position and color of mucous membranes) and individual symptoms such as tremors, diarrhea, convulsions, drowsiness, etc.

### 4.6. Hepatoprotective Activity

The study was performed on the model of acute tetrachloromethane hepatitis [39,40,41]. Research in the hepatoprotective activity of the cranberry-leaves extract (E1) and its preparations (E2-E8) with amino acids (valine, arginine, alanine, glycine, histidine, aspartic acid, and taurine) was carried out at the Clinical and Biological Experimental Base of IFNMU in accordance with the National “General Ethical Principles of Animal Experiments” (Ukraine, 2001), which correspond to the provisions of the “European Convention for the Protection of Vertebrate Animals Used for Experimental and Other Scientific Purposes” (Strasbourg, 1986) [42,43,44] and was approved by the Ethics Commission of Ivano-Frankivsk National Medical University (#133/23 dated 29 March 2023).

The research in the hepatoprotective activity of the studied extracts was carried out on 66 white non-linear sexually mature rats grown in the kennel of the Clinical and Biological Experimental Base of IFNMU weighing 160–240 g, which were standardized according to physiological and biochemical indicators and divided into 11 groups of 6 animals each: 1 group—intact animals; 2nd group—control pathology; 3–10 groups—animals which consumed the cranberry-leaves extract and its preparation with amino acids at a dose of 25 mg/kg (bw); Group 11—animals that received the referent drug “Silibor” at a dose of 25 mg/kg (bw) [38]. The laboratory animals were kept in accordance with the current “Sanitary rules regarding the arrangement, equipment, and maintenance of experimental biological clinics (vivariums)” at a temperature of 18–20 °C and relative humidity of 50–55%. They were fed a full-fledged diet, according to a standard scheme, with free access to water.

To study hepatoprotective activity, liver damage in the rat groups was caused by a 50% tetrachloromethane oil solution in a dose of 0.8 mL per 100 g (bw) for 2 days in 24 h. The experimental animals consumed aqueous solutions of the studied extracts at a dose of 25 mg/kg (bw). The preparation “Silibor” (PhC “Zdorovya”, Kharkiv, Ukraine) was used as a referent medicine. “Silibor” tablets, after removing the shells, were crushed in a mortar and administered intragastrically in the form of a 1% starch suspension. The intact animals consumed purified water. The studied extracts and the comparison drug were consumed by the rats for 1 h and 2 h after taking the hepatotropic poison.

The rats were decapitated after ingestion of the first tetrachloromethane dose on the third day. The results were calculated on the basis of biochemical and functional indicators of the liver state, which were determined within 24 h after the last dose of tetrachloroethane. Liver weight coefficients were calculated in a percentage, as the ratio of liver and body weights.

The study of biochemical indicators was carried out on the basis of the Center of Bioelementology IFNMU (certificate of technical competence No. 037/19 from 13 June 2019 to 12 June 2024).

The assessment of the intensity of the peroxidic destructive transformations in the animals’ bodies was determined by the content of TBA-reactants in the blood serum and liver homogenate. The effectiveness of the hepatoprotective effect of the extracts was evaluated by changes in the level of alanine aminotransferase (AlAt) and aspartate aminotransferase (AsAt) in blood serum, which are hepatospecific markers of cytolysis. The activity of cytolysis enzymes AlAt and AsAt was measured in blood serum using the Reitman-Frenkel method using the standard set of reagents from the company “SIMKO Ltd.” (Dnipro, Ukraine) [38,39,40].

The total level of lipid peroxidation (TLLP) was determined with 2-thiobarbituric acid (TBA) by the spectrophotometric method according to the E.N. Korobeinikova method using the biochemical set of the “Filisit-Diagnostika” company (Dnipro, Ukraine).

The hepatoprotective activity of the studied extracts is evidenced by the survival rate of the animals, the liver weight ratio, and the normalization of blood serum biochemical parameters and liver homogenate [38].

### 4.7. Statistical Analysis

The mean and standard deviation (SD) of the samples were calculated according to the State Pharmacopoeia of Ukraine, the monograph “Statistical Analysis of the Results of a Chemical Experiment” [33]. The average value was established on the basis of 5 measurements. The confidence interval is usually at the 95% significance level. Using Student’s criterion limit, values of the confidence interval were calculated. The data are presented as the mean ± SD. The level of statistical significance was not more than *p* < 0.05 [17,33,34].

## 5. Conclusions

The chemical composition and hepatoprotective activity of the American cranberry-leave extract and its seven amino-acids preparations were studied.

The researchers identified 19 phenolic substances in the American cranberry-leaves extracts and their contents were determined by the HPLC-PDA method. There were eight flavones and flavonols, four anthocyanins, three hydroxycinnamic acids, and two catechins in the extracts studied. Quercetin glycosides predominate in all extracts. Hyperoside, avicularin, and quercitrin were dominant among the individual substances. The main amino acids (arginine, aspartic acid, glycine, histidine, taurine, and valine) were identified and quantified by the UPLC-MS/MS method.

The administration of the extracts from the American cranberry leaves on rats resulted in a decrease in the lipid-peroxidation process in the experiment on acute toxic damage by tetrachloromethane. The most promising hepatoprotective effect has been established for the cranberry extracts prepared with valine and arginine.

## Figures and Tables

**Table 1:** Composition of phenolic substances in the American cranberry-leaves extracts determined by HPLC-PDA method.

Phenolic Compounds	Content in the Extract, µg/g *							
	E1	E2	E3	E4	E5	E6	E7	E8
Cyanidin-3-*O*-galactoside	ND	NQ	ND	ND	NQ	NQ	54 ± 9	105 ± 6
Cyanidin-3-*O*-arabinoside	ND	NQ	ND	ND	NQ	NQ	129 ± 11	210 ± 4
Hyperoside	16,125 ± 307	6848 ± 67	908 ± 6	8044 ± 104	10,705 ± 109	6407 ± 241	5279 ± 20	5604 ± 13
Isoquercitrin	1167 ± 35	399 ± 4	171 ± 5	480 ± 15	667 ± 2	397 ± 11	339 ± 4	404 ± 9
Reynoutrin	924 ± 64	1586 ± 15	188 ± 5	2007 ± 9	2542 ± 21	1499 ± 61	1084 ± 3	0760 ± 14
Quercetin-3-*O*-arabinopyranoside	5821 ± 58	2414 ± 23	243 ± 11	2903 ± 56	3911 ± 46	2306 ± 54	1801 ± 5	1548 ± 15
Avicularin	12,860 ± 407	3084 ± 27	595 ± 8	5930 ± 119	6446 ± 54	1712 ± 69	707 ± 14	67 ± 3
Quercitrin	12,333 ± 361	4703 ± 39	477 ± 15	5569 ± 113	7541 ± 87	4655 ± 102	3777 ± 6	3858 ± 36
Quercetin	952 ± 0.067	2823 ± 46	103 ± 8	717 ± 10	2612 ± 67	4153 ± 107	4096 ± 11	6992 ± 42
Kaempferol-3-*O*-rhamnoside	351 ± 13	110 ± 2	4 ± 1	175 ± 2	196 ± 5	111 ± 9	117 ± 2	119 ± 1
Kaempferol	50 ± 2	54 ± 1	9 ± 1	14 ± 1	52 ± 2	68 ± 3	49 ± 1	82 ± 1
Chlorogenic acid	2356 ± 35	1117 ± 15	133 ± 3	685 ± 12	1599 ±46	1222 ± 26	657 ± 10	672 ± 2
Neochlorogenic acid	2497 ± 108	720 ± 17	47 ± 1	687 ± 6	1733 ± 64	1093 ± 17	687 ± 10	735 ± 21
4-*O*-caffeoylquinic acid	1100 ± 45	834 ± 19	175 ± 1	649 ± 31	913 ± 8	863 ± 25	208 ± 2	235 ± 5
*p*-Coumaric acid	136 ± 10	70 ± 1	11 ± 1	17 ± 1	93 ± 6	71 ± 3	30 ± 2	16 ± 1
(+)-Catechin	911 ± 42	415 ± 7	509 ± 2	666 ± 16	758 ± 6	484 ± 82	391 ± 23	383 ± 4
(–)-Epicatechin	247 ± 3	1407 ± 55	190 ± 17	2147 ± 97	2166 ± 59	1540 ± 60	886 ± 12	444 ± 9
Procyanidin A1	1313 ± 26	1007 ± 69	139 ± 7	026 ± 67	1398 ± 93	1209 ± 36	447 ± 16	297 ± 19
Procyanidin A2	2053 ± 32	1689 ± 25	69 ± 3	2823 ± 6	4047 ± 74	2708 ± 49	1303 ± 16	902 ± 7
Total amount (mg/g)	64,196	29,280	3971	34,539	47,379	30,498	22,041	23,433

Notes: * Results are expressed as µg/g for dry weight in the case of extracts E1, E2, E5, and E6, and for viscous masses in the case of extracts E3, E4, E7, and E8. ND—not detected, NQ—not quantified (amount below LOQ).

**Table 2:** LWC of the experimental animals.

Group #	Group of Experimental Animals	m _animal_,	m _liver_	LWC
1	Intact animals	174.2 ± 11.7	6.1 ± 0.4	3.5 ± 0.1
2	Control pathology (CCl4)	235.0 ± 15.9	12.0 ± 0.6	5.1 ± 0.5
3	E1	210.0 ± 9.4	8.9 ± 0.7	4.2 ± 0.2
4	E2	233.3 ± 8.6	8.9 ± 0.6	3.8 ± 0.2
5	E3	218.3 ± 12.3	8.1 ± 0.7	3.7 ± 0.2
6	E4	221.7 ± 12.3	9.3 ± 0.9	4.2 ± 0.3
7	E5	191.7 ± 7.9	7.9 ± 0.6	4.1 ± 0.2
8	E6	210.0 ± 9.6	8.4 ± 0.5	4.0 ± 0.2
9	E7	225.0 ± 11.0	9.2 ± 1.1	4.1 ± 0.3
10	E8	226.7 ± 12.7	9.2 ± 0.7	4.1 ± 0.1
11	Silibor	216.7 ± 6.4	8.6 ± 0.5	3.9 ±0.1

**Table 3:** The effect of the American cranberry-leaves extracts on the course of acute toxic hepatitis caused by tetrachloromethane (M ± m).

#	Group of Animals	Biochemical Indicators			
		Blood Serum			Liver Homogenate
		AlAt, μmol/h.mL	AsAt, μmol/h.mL	TBA-Reactants nmol/mL	TBA-Reactants nmol/mL
1	Intact animals	0.76 ± 0.05	0.96 ± 0.06	3.23 ± 0.12	2.45 ± 0.09
2	Control pathology (CCl4)	2.56 ± 0.11 *	2.32 ± 0.11 *	5.96 ± 0.28 *	6.15 ± 0.26 *
3	E1	1.28 ± 0.04 */**	1.36 ± 0.06 */**/#	4.21 ± 0.20 */**/#	3.64 ± 0.13 */**
4	E2	0.96 ± 0.04 */**/#	1.09 ± 0.05 */**/#	3.52 ± 0.16 */**/#	2.90 ± 0.14 */**/#
5	E3	0.85 ± 0.06 */**/#	1.04 ± 0.052 */**/#	3.38 ± 0.12 */**/#	2.76 ± 0.15 */**/#
6	E4	1.31 ± 0.05 */**	1.34 ± 0.05 */**/#	4.15 ± 0.21 */**/#	3.78 ± 0.17 */**/#
7	E5	1.26 ± 0.06 */**	1.30 ± 0.05 */**/#	4.01 ± 0.22 */**	3.80 ± 0.17 */**/#
8	E6	1.06 ± 0.06 */**/#	1.16 ± 0.06 */**/#	3.71 ± 0.16 */**/#	3.24 ± 0.17 */**/#
9	E7	1.21 ± 0.05 */**	1.28 ± 0.07 */**	3.97 ± 0.26 */**	3.65 ± 0.17 */**
10	E8	1.10 ± 0.04 */**/#	1.12 ± 0.05 */**/#	3.80 ± 0.17 */**/#	3.10 ± 0.17 */**/#
11	Silibor	1.27 ± 0.06 */**	1.24 ± 0.05 */**	3.90 ± 0.16 */**	3.57 ± 0.16 */**

Notes: AlAt: alanine aminotransferase, AsAt: aspartate aminotransferase, TBA-reactants: 2-thiobarbituric acid reactants. *—the reliability of the deviation in relation to the data of the intact animals group (*p* ≤ 0.05). **—the reliability of the deviation in relation to the data of the control pathology group (*p* ≤ 0.05). #—the reliability of the deviation in relation to the data of the comparison drug “Silibor” group (*p* ≤ 0.05).

## Supplementary Materials

The following supporting information can be downloaded at: https://www.mdpi.com/article/10.3390/plants12102010/s1, Figure S1: Typical UPLC-MS/MS chromatograms of phenolic components in the samples; Figure S2: Comparative HPLC-PDA profiles at 325 nm of different extracts.
