# Supplementary material for: American Cranberry (Oxycoccus macrocarpus (Ait.) Pursh) Leaves Extract and Its Amino-Acids Preparation: The Phytochemical and Pharmacological Study
Source: Plants (Basel). 2023 May 17;12(10):2010. doi: 10.3390/plants12102010 (PMC10221213; doi:10.3390/plants12102010)
Supplement: Supplementary file 1 [file plants-12-02010-s001.zip › plants-2381432-SI.pdf]

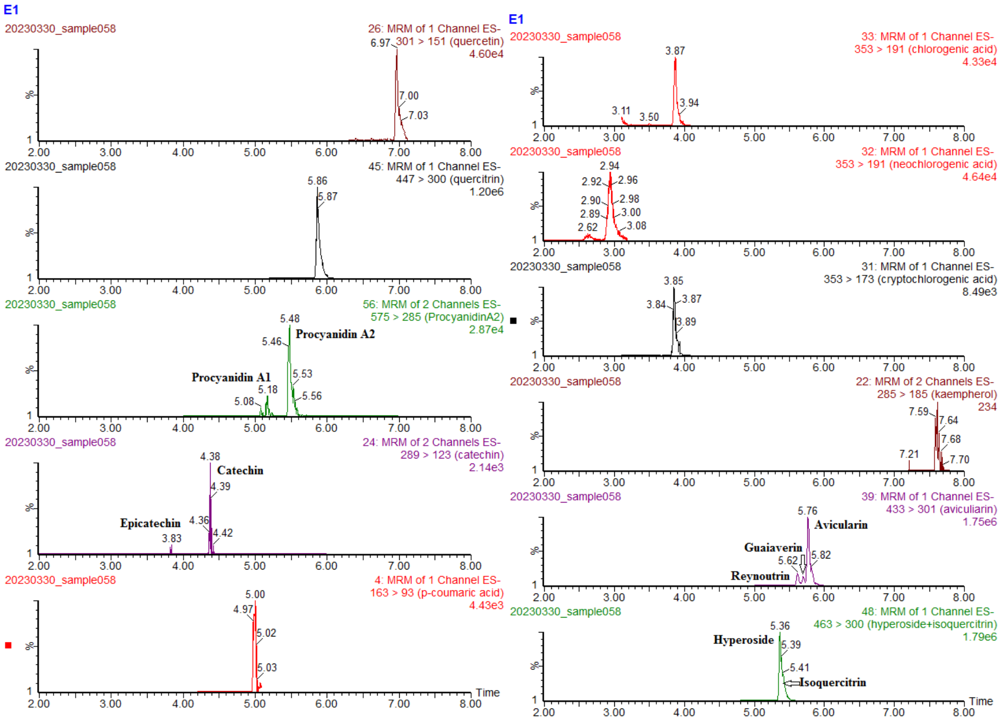

Figure S1. Typical UPLC-MS/MS chromatograms of phenolic components in the samples.

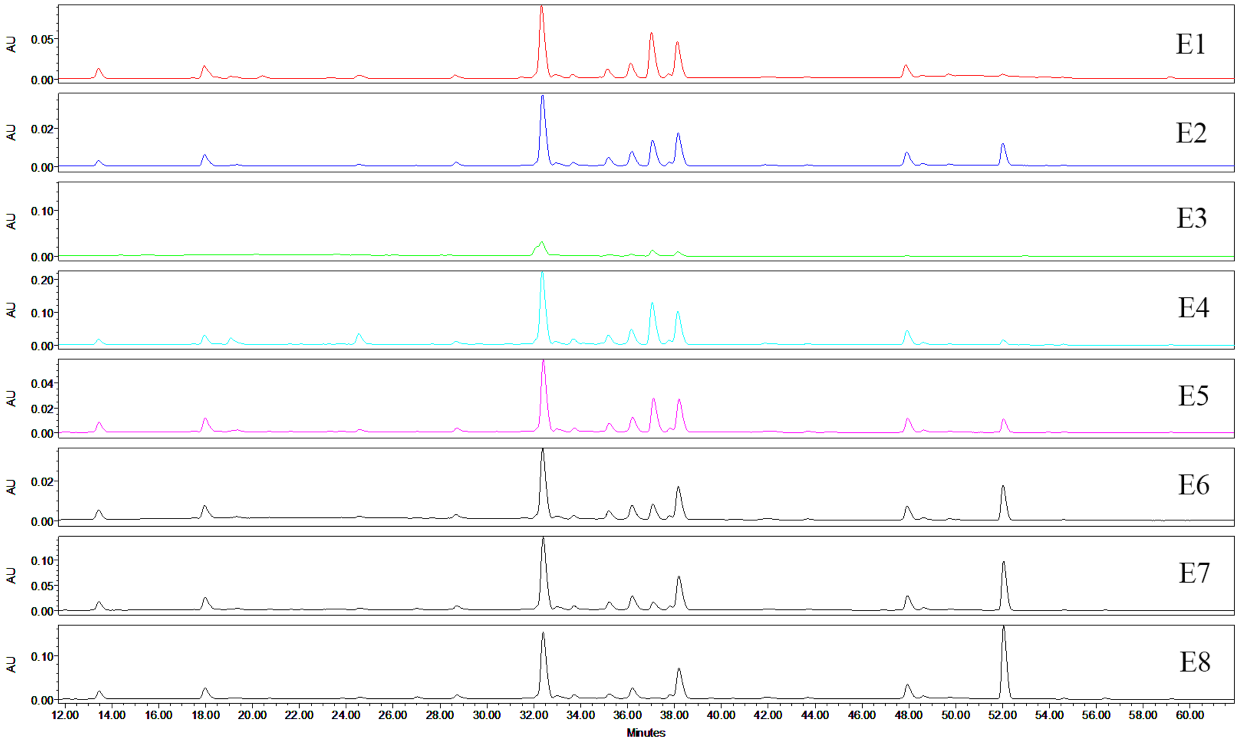

Figure S2. Comparative HPLC-PDA profiles at 325 nm of different extracts.
